# The impact of corrective exercises, kinesiology taping, and mechanical correction on pain and foot shape in women with hallux valgus

**DOI:** 10.3389/fphys.2025.1473278

**Published:** 2025-04-07

**Authors:** Agnieszka Jankowicz-Szymańska, Katarzyna Wódka, Eliza Smoła, Marta A. Bibro

**Affiliations:** Faculty of Medicine and Health Sciences, University of Applied Sciences in Tarnow, Tarnow, Poland

**Keywords:** hallux valgus, pain, women, physiotherapy, conservative treatment

## Abstract

**Background:**

This study aimed to assess the effect of corrective exercises and exercises supplemented by kinesiology taping or an orthosis on pain and foot alignment in women with hallux valgus (HV).

**Methods:**

Eighty-two women with HV were randomly divided into groups: E (n = 24) who exercised for 12 weeks; EKT (n = 18) who exercised and used kinesiology taping on HV; EMC (n = 15) who performed exercise and used an orthosis to correct their big toe position; and CHV (25 women with HV without therapy). Additional control group C: (n = 31) women with normal hallux. The Wejsflog index, Clarke’s angle, alpha and beta angles, and pain intensity in the big toe area were assessed at the beginning and after therapy.

**Results:**

The Wejsflog index was significantly lower in women with HV and increased significantly after therapy in the E and EKT groups. Clarke’s angle did not differ between women with and without HV, and Clarke’s angle did not change after therapy. The HV angle decreased significantly in groups E and EKT but was still significantly greater than that in group C. The fifth toe varus angle did not differ between women with and without HV and decreased significantly in the right foot after therapy in group E. Pain in the HV area decreased significantly in all groups undergoing therapy.

**Conclusion:**

Women with HV have a greater forefoot width, but their longitudinal arch and fifth toe position do not differ. Exercises significantly reduce pain and improve hallux alignment. Combining exercises with kinesiology taping or an orthosis does not increase the therapeutic effect. Although the observed effects of conservative therapy are promising, it should be remembered that long-term effects have not been studied.

**Trial registration:**

The Australian New Zealand Clinical Trials Registry (ACTRN12621000902897).

## Background

The issue of foot pathology is a problem widely discussed in the literature in relation to the population of both children (Knörr et al., 2022; Woźniacka et al., 2015) and adults (Okamura et al., 2019; Bac et al., 2022; Pita-fernandez et al., 2017). One of the most common foot health problems in adults is hallux valgus (Omae et al., 2021). Hallux valgus is a deformity in which the first toe is adducted at the metatarsophalangeal joint with an accompanying displacement of the first metatarsal bone so that the head of the metatarsal bone shifts medially and forms a more or less visible bulge on the medial side of the forefoot (Menz et al., 2006; Mickle et al., 2009; Nguyen et al., 2010). Lalevee et al. found that there is a relationship between isolated HV, isolated flatfoot, or combined HV-flatfoot and increase pronation in the first metatarsal bone. They also observed that isolated HV or combined HV-flatfoot correlates with greater tarsometatarsal pronation and naviculocuneiform supination, compared with isolated flatfoot, and normal foot (Lalevee et al., 2025). The presence of hallux valgus reduces quality of life because it is associated with pain in the hallux area, metatarsalgia, a limited range of motion in the metatarsophalangeal (MTP) and interphalangeal (IP) joints of the hallux, and increased tension in the calf muscles (Wang et al., 2022; Chen et al., 2022; Jankowicz-Szymańska et al., 2022). As a consequence of these changes, foot function is impaired, balance deteriorates, and the risk of falling even doubles (Sánchez-Sanjuan et al., 2022). Hallux valgus is diagnosed in every fourth person aged between 18 and 65 years, and is diagnosed slightly more often in every third person over 65 years of age (Nix et al., 2010). Hallux valgus in older adult people is associated with impaired function of the calf muscles during gait, which is compensated by increased abduction of the hip joint (Liu et al., 2025). This deformity is one of the most common causes of foot dysfunction and occurs mainly in women (Li et al., 2022). Some studies indicate a relationship between the occurrence of hallux valgus and body mass index (BMI) and the type of footwear worn. However, the mechanism underlying the development of this foot defect has not been fully explained. The impact of the mentioned risk factors, as well as of degenerative changes in the knee joint, hindfoot valgus or the length of the first metatarsal bone on hallux valgus, has not been clearly confirmed (Yamashita et al., 2023; Żłobiński et al., 2021). Most likely, the etiology of hallux valgus is multifactorial with a significant genetic component (Ray et al., 2019; Okuda et al., 2014).

The primary goal of hallux valgus therapy is to reduce pain, which affects 76%–83% of people with this deformity (Nix et al., 2012; Buldt et al., 2015), and to improve the positioning of the first toe. One of more than 100 surgical treatment methods or conservative treatments are used (Robinson and Limbers, 2005; Gądek and Liszka, 2013). A literature review revealed that the most frequently used methods for conservative treatment are exercises, kinesiology taping, orthoses and corrective inserts.

The most frequently recommended exercises include strengthening the short plantar muscles, including the abductor hallucis and flexor hallucis brevis. Using electromyography, Arinci İncel et al. (2003) showed that in people with hallux valgus, the activity of these muscles is lower than that of their antagonists. The effectiveness of toe-spread-out exercises and short foot exercises has been documented by Kim et al. (2013). In the treatment of hallux valgus, exercises are also used to correct the position of the hindfoot, increase the flexibility of shortened muscles and improve the mobility of the foot joints, particularly of the first toe, as well as proprioceptive exercises (Arge et al., 2012; Kim M-H. et al., 2015; Glasoe, 2016). It is believed that simple exercises performed at home (twice a day for 15 min), even if they do not change the position of the first toe, may be effective in reducing pain and improving the range of motion in the first MTP joint (Arge et al., 2012).

Kinesiology taping in patients with hallux valgus is also used, primarily to correct the alignment of the hallux and reduce pain. There are many reports on the effectiveness of kinesiology taping; however, the technique for applying this tape has not been clearly established (Tantawy et al., 2019; Biz et al., 2022; Bac et al., 2020a; Nunes et al., 2021). Many techniques have been proposed in the literature, including the use of rigid, not just elastic, tape (Bayar et al., 2011; Radwan et al., 2017). It is most commonly recommended to apply a Y-shaped elastic tape. The end of the tape was applied to the base of the big toe, and the Y arms were placed with a slight to moderate stretch around the passively corrected big toe. Correction of the hallux alignment should involve abduction and derotation, without traction (Bayar et al., 2011). Recommendations on how long to apply the tape also vary. Lee and Lee (2016) observed that the alignment of the big toe improved and pain was reduced after 3 months of kinesiology taping, while Jeon et al. (2004) observed this after just 4 weeks. The study by Żłobiński et al., 2021) shows that even immediately after the application of kinesio taping, the hallux valgus angle is reduced, the position of the hindfoot improves and the foot load is normalized, which can be seen on a baropodometric platform (in the discussed studies, an elastic tape with a tension of 75%–100% was used: first, a Y-shaped tape was applied as described above, but with the long Y arm extended to the calcaneus, followed by an I-shaped tape from the medial edge of the first metatarsal to the lateral edge of the fifth metatarsal to improve the stability of the first metatarsal).

After exercises and kinesiology taping, orthoses are the third most frequently used method for correcting hallux valgus. Biomechanical orthoses, which work by passively abducting the big toe and keeping the pathologically shortened muscles, ligaments, and tendons in a stretched position, are most often recommended. Typically, these orthoses allow the strength and range of abduction to be adjusted to the patient’s needs but are not comfortable and only suitable for use at night. In contrast, gel separators can be used all day long, are light and comfortable, but are less effective (Li et al., 2022). Gel separators are also rated lower than inserts (Dissaneewate et al., 2022). Separators, like orthoses, can be used as part of conservative treatment, but also to maintain the effects of surgical intervention (Krześniak and Truszczyńska-Baszak, 2024; Colò et al., 2024). Arvanitakis et al. also propose the Innovative Hallux Valgus Sock. According to the authors, the sock is comfortable to use, can be hidden discreetly in footwear, reduces pain, normalizes muscle function (EMG study), and improves hallux positioning (photographic measurement) (Arvanitakis et al., 2024). Although most studies confirm the analgesic effect of corrective orthoses, changes in the position of the first toe are not always observed (Plaass et al., 2019; Nakagawa et al., 2019; Dygut et al., 2020).

All the methods described affect the symptoms, not the causes, of hallux valgus, and although they do not completely eliminate the deformation, they often provide satisfactory results. These methods are used together or as stand-alone therapies, and the methods that produce the best results have not yet been identified. This study aimed to compare the effectiveness of exercises combined with kinesiology taping or exercises combined with mechanical correction for the hallux valgus treatment. The experience and research results of the authors cited above helped us optimally plan exercises and choose the method for applying kinesiology tape and the type of orthosis. We assumed that exercise should be the basic therapy method because once patients learn about it, they can use it forever and without restrictions. Moreover, we wanted to create internal motivation in our patients, give them a sense of agency, and convince them to take responsibility for their health and wellbeing (Deci and Ryan, 2008). We assumed that the basic determinants of the effectiveness of therapy were the reduction in pain intensity, the hallux valgus angle and the fifth toe angle. We also observed changes in the size of the medial longitudinal and distal transverse arches of the foot because their shape and importance in the development of hallux valgus are still controversial.

The following research questions were tested:

1. Is hallux valgus in women associated with changes in the height of the longitudinal and transverse arches of the foot?
2. Can conservative treatment reduce pain in women with hallux valgus?
3. Can conservative treatment improve foot alignment in women with hallux valgus?
4. Can supplementing the corrective exercise program with kinesiology taping or an orthopedic device influence the therapeutic effect?

The following research hypotheses were formulated:

1. Hallux valgus in women is associated with changes in the height of the longitudinal and transverse arch of the foot.
2. Conservative treatment can reduce pain in women with hallux valgus.
3. Conservative treatment can improve foot alignment in women with hallux valgus.
4. Supplementing the corrective exercise program with kinesiology taping or an orthopedic device can affect the therapeutic effect.

## Methods

### Characteristics of the experimental group

In total, 164 women responded to the invitation. In the first step, the volunteers (all of whom responded to the invitation published on the internet and broadcast on local radio) were acquainted with the project’s purpose and plan in detail, and whether they met the inclusion conditions was checked. Twelve women were excluded.

The exclusion criteria from the study were as follows: age under 35 years (the limit of early adulthood), excessive pronation of the feet, advanced flat feet, surgical correction of hallux valgus, injury to the lower limb or pelvis up to 2 months before the start of the study, neurological disease, diabetes, gout and rheumatoid conditions. All women, including those who did not meet the inclusion criteria, could benefit from foot assessment and advice from a physiotherapist. In the next step, the shape of the feet of 152 women aged 38–58 years was assessed using the Manchester scale (Garrow et al., 2001). A total of 102 women had hallux valgus of mild or moderate deformity in one or both feet, and 50 women had correctly positioned hallux in both feet. Women with hallux valgus were randomly divided into four groups (this was done after the first series of tests were completed), and 50 women with properly shaped feet were classified into the additional control group. All the women signed consent to participate in the study. During the study, 39 women resigned from participating. The reasons for resignation were random events in the family, the patient’s illness, and attendance at meetings less than 90%.

Ultimately, group E (exercises) included 24 women who performed corrective exercises under the supervision of an experienced therapist twice a week for 45 min for 12 weeks. At home on the remaining 5 days of the week, they performed five exercises prescribed by the therapist.

The EKT group (exercises, kinesiology taping) comprised 18 women who, in addition to exercising, had kinesiology tape applied to the hallux valgus, which was changed every 4 days.

Group EMC (exercises, mechanical correction) was composed of 15 women who, in addition to exercising, underwent mechanical correction of hallux valgus every night using an orthopedic device that works by keeping the hallux in an abducted position (MARCIN type device, manufactured by NEURON s.c.).

The control group CHV (control group with hallux valgus) consisted of 25 women with hallux valgus who were not subjected to any therapy.

**FIGURE 1:**
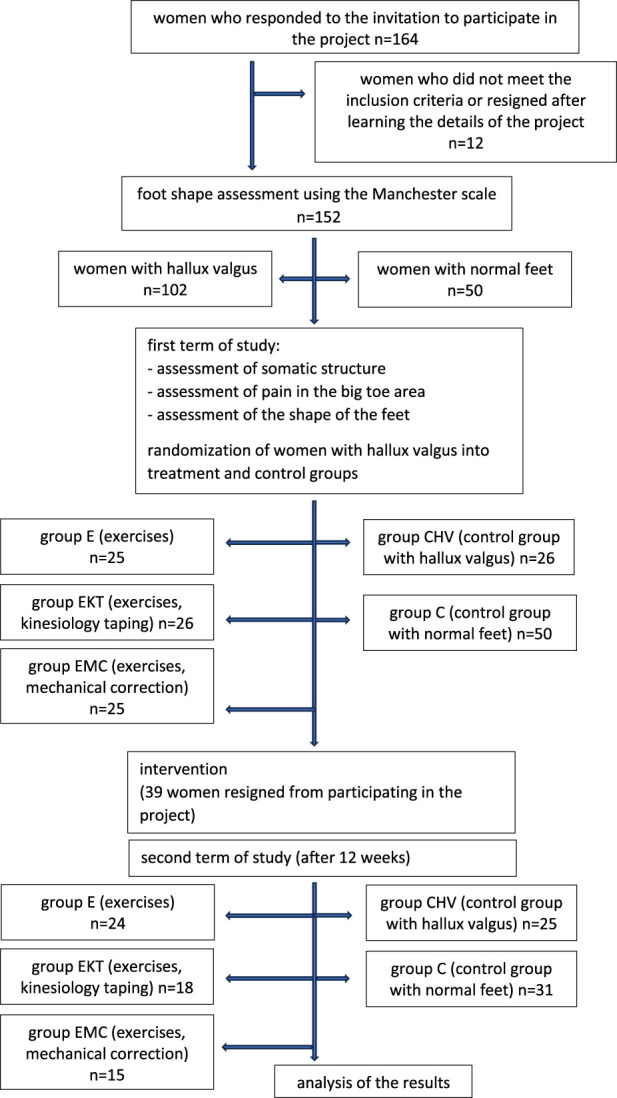
Study scheme.

Additionally, a control group C (31 women) was created. It was composed of women, including volunteers who responded to the advertisement, with properly shaped feet who felt no pain in their feet. Women from this group did not practice any sports discipline and were not subjected to any therapy during the project or in the three preceding months (information from the interviews with the patients) (Figure 1).

Approval to conduct the study was obtained from the Regional Medical Chamber (No. 4/0177/2016), and the study was registered in the Australian New Zealand Clinical Trials Registry (ACTRN12621000902897). All the requirements of the Declaration of Helsinki for research involving human participants were respected during the study.

#### Improvement programme with a therapist

• introductory part (5–10 min) – warm-up exercises;
• main part (30 min) – sample exercises: active flexion and extension of the ankle-shin joint in a sitting position, circling the feet in a sitting position, rolling a tennis ball with the foot while standing, passive and active flexion and extension of the metatarsophalangeal joints in a sitting position, passive and active toe abduction in a sitting position, active hallux extension in a sitting position, short foot exercises in sitting and standing positions, toe curling in a sitting position, foot exercises with a loop band, and exercises to increase the flexibility of the gastrocnemius and soleus muscles; additionally, exercises were conducted on unstable surfaces (e.g., standing with both feet on a sensorimotor disc) because research indicates that this type of training is beneficial to women (Matla et al., 2021);
• final part (5 min) – relaxation and breathing exercises.

#### Exercises performed at home

Study participants were asked to perform the following exercises at home: rolling a tennis ball with the foot, stretching a loop band hooked to the big toes of the right and left feet, autotraction in the first metatarsophalangeal joint, short foot exercise, active abduction of the toes and hallux (women were obliged to keep a diary in which they recorded how regularly they performed the exercises; analysis of diary entries showed that the regularity of performing home exercises ranged from 60% to 95%).

#### Method for applying kinesiology tape

Kinesiology tape was changed for each woman from the EKT group every 4 days for 12 weeks. The first application was performed immediately after the first exercises with the therapist. The Y form was applied via the fascial technique (15%–25%). The base of the tape was applied to the big toe, the tails were glued along the medial edge of the foot, and then fascial correction was performed in the I form. The base was glued on the medial dorsal surface of the forefoot, and the tail was attached toward the outer dorsal surface of the foot. While applying the tape, the foot was placed in a flexed position. Additionally, if the big toe was in rotation, an application to correct the position of the toe was added before the procedure was performed.

### Research tools

#### Assessment of somatic structure

Body structure was assessed once. Body height was measured using a calibrated anthropometer (Alumet, Warsaw, Poland) with an accuracy of 0.01 m. The body weight of the examined women was estimated with an accuracy of 0.1 kg using a TANITA BF 350 scale (Tanita Corporation of America Inc.). Based on these data, BMI was calculated according to a standard formula.

#### Assessment of pain in the big toe area

Women with hallux valgus were asked twice (at the beginning of the project and after 12 weeks) about the level of the greatest pain they felt in the hallux area (right or left) during the previous 7 days. The NRS was used (Ferreira-Valente et al., 2011).

#### Assessment of the shape of the feet

All women participating in the project, regardless of which group they were in, underwent two computer assessments (before and after the completion of the rehabilitation programme, i.e., after 12 weeks) of the plantar side of the feet using a CQ Elektronik podoscope (CQ Elektronik System).

The hallux valgus angle α [°] was determined as the angle between the tangent to the medial edge of the foot and the tangent drawn from the point at the widest part of the forefoot to the outer edge of the hallux. The norm for the hallux valgus angle is 0°–9° (Bac et al., 2020b).

The little toe varus angle β [°] was determined as the angle between the tangent to the outer edge of the foot and the tangent drawn from the point at the widest part of the forefoot to the outer edge of the little toe. The norm for the hallux valgus angle is 0°–5° (Bac et al., 2020b).

Foot length [mm] and foot width [mm] were assessed, and the Wejsflog index was calculated (the proportion of foot length to foot width, where index values closer to 3 indicate a proper transverse arch and values closer to 2 indicate transverse flat feet). It was assumed that Wejsflog index values of 2.50–3.00 indicate a normal transverse arch, while values of 2.49 or less indicate transverse flat feet (Bac et al., 2020b).

The size of the longitudinal arch was examined using Clarke’s angle [°] (the angle between the tangent to the medial edge of the footprint and the line connecting the point of the largest depression and the contact of the medial tangent with the edge of the forefoot). It was assumed that values ranging from 0° to 41° indicated a reduced longitudinal arch (flat foot), values ranging from 42° to 54° indicated a properly arched foot, and values of 55° and above indicated a hollow foot (Bac et al., 2020b).

Data analysis was performed using Statistica ver. 13.0 software. Among the methods used were descriptive statistics, the Shapiro‒Wilk test (for normality of distribution), the F test (for homogeneity of variance), Tukey’s HSD test (to compare several groups of quantitative variables assuming normality of distribution), and the Kruskal‒Wallis test (to compare several groups when the variables were not normally distributed or the variances were not homogeneous). To verify intragroup variability (the same parameters were measured twice), the nonparametric Wilcoxon pairwise order test (when the distributions did not comply with the normal distribution) or Student’s t-test for dependent samples (when the distributions of variables complied with the normal distribution) were used. The significance level adopted was α < 0.05.

## Results

The examined women were aged 38–58 years. The youngest women were in group C (47.03 ± 4.61 years), and the oldest were in group EKT (51.00 ± 6.75 years). Age did not significantly differentiate the studied groups. Women in group C had the lowest BMI (23.96 ± 3.45 kg/m^2^), and women in group CHV had the highest BMI (28.17 ± 0.42 kg/m^2^). BMI differed significantly between groups C and CHV (p < 0.05). No significant differences in BMI were noted between the remaining groups.

In the first examination, the level of pain in the big toe area was greater in the E, EKT and EMC groups than in the CHV group. For group E, this difference was statistically significant. The therapy significantly reduced the average pain experienced in all groups: in group E, by 2.67 points on the NRS; in group EKT, by 2.78 points; and in group EMC, by 2.73 points. However, it was noted that in eight people, the pain remained unchanged or worsened (four people from group E, three from group EKT and one from group EMC), which explains the difference between the mean and median and the high standard deviation value in the second examination. At the second examination, no differences between the groups were noted (Table 1).

**TABLE 1:** Pain intensity in the area of hallux valgus in women under research before and after therapy.

Pain intensity	Group	1st term of study				2nd term of study				p^1st & 2nd^ (EF)
		x̅ (CI)	SD	Me (Q1-Q3)	Min-Max	x̅ (CI)	SD	Me (Q1-Q3)	Min–Max	
The greatest pain experienced in the last week	E	4.75 (3.54–5.95)	2.86	5.00 (4.00–6.50)	0.00–10.00	2.08 (1.02–3.13)	2.50	0.50 (0.00–4.00)	0.00–7.00	p < 0.01* (1.39)
	EKT	4.39 (3.23–5.53)	2.31	5.00 (4.00–6.00)	0.00–7.00	1.61 (0.56–2.66)	2.11	0.25 (0.00–3.00)	0.00–7.00	p < 0.01* (1.96)
	EMC	4.03 (2.45–5.61)	2.86	5.00 (0.00–6.00)	0.00–8.00	1.33 (0.19–2.47)	2.06	0.00 (0.00–4.00)	0.00–5.00	p = 0.01* (1.64)
	CHV	1.76 (0.80–2.71)	2.31	5.00 (0.00–4.00)	0.00–6.00	2.62 (1.48–3.75)	2.75	2.00 (0.00–6.00)	0.00–8.00	p = 0.08 (0.55)
	E & EKT p = 1.00; E & EMC p = 1.00 E & CHV p < 0.01*; EKT & EMC p = 1.00 EKT & CHV p = 0.05; EMC & CHV p = 0.21					E & EKT p = 1.00; E & EMC p = 1.00 E & CHV p = 1.00; EKT & EMC p = 1.00 EKT & CHV p = 1.00; EMC & CHV p = 1.00				

CI–confidence interval; EF - Effect Size dppc2 sensu Morris (Morris, S. B. (2008). *Estimating Effect Sizes from Pretest-Posttest-Control Group Designs*. organizational research methods, 11 (2), 364–386. http://doi.org/10.1177/1094428106291059).

E–women with hallux valgus doing corrective exercises; EKT, women with hallux valgus doing corrective exercises and wearing Kinesio tape; EMC, women with hallux valgus performing corrective exercises and using an orthosis at night; CHV, control group, women with hallux valgus; * - statistically significant difference.

The α angle at the first examination was significantly smaller in group C than in all the other groups. Under the influence of therapy, the α angle decreased in group E (by 2.83° in the right foot and by 2.31° in the left foot) and in group EKT (by 2.74° in the right foot and by 1.99° in the left foot). These changes were statistically significant. However, in the second examination, in both groups, the α angle was still significantly greater than that in group C (Table 2).

**TABLE 2:** Hallux valgus angle (α) in the right and left feet of the women under study before and after therapy.

Foot	Group	1st term of study				2nd term of study				p^1st & 2nd^ (EF)
		x̅ (CI)	SD	Me (Q1-Q3)	Min-Max	x̅ (CI)	SD	Me (Q1-Q3)	Min–Max	
Right	E	23.56 (20.19–26.93)	7.98	22.70 (18.50–27.70)	6.30–36.00	20.73 (17.78–23.67)	6.97	19.35 (15.70–24.30)	7.90–35.70	p < 0.01* (0.51)
	EKT	22.99 (18.98–27.01)	8.07	23.30 (18.70–26.60)	1.50–37.80	20.25 (16.26–24.24)	8.03	20.25 (17.40–23.70)	1.50–34.80	p < 0.01* (0.53)
	EMC	21.74 (18.32–26.31)	6.86	22.80 (17.90–28.50)	7.00–31.70	21.99 (18.56–25.42)	6.20	22.50 (17.90–27.70)	10.20–32.60	p = 0.69 (0.05)
	C	6.49 (5.83–7.24)	1.87	7.10 (6.00–7.70)	0.40–8.60	6.49 (5.71–7.28)	2.11	7.10 (6.10–7.90)	0.80–9.00	p = 0.63 (0.01)
	CHV	17.86 (15.21–20.97)	7.05	17.50 (14.20–22.00)	6.20–32.10	17.80 (14.84–20.49)	6.93	16.40 (13.60–20.60)	6.50–32.10	p = 0.62 (0.01)
	E & EKT p = 1.00; E & EMC p = 1.00 E & C p < 0.01*; E & CHV p = 0.37 EKT & EMC p = 1.00; EKT & C p < 0.01* EKT & CHV p = 0.65; EMC & C p < 0.01* EMC & CHV p = 1.00; C & CHV p < 0.01*					E & EKT p = 1.00; E & EMC p = 1.00 E & C p < 0.01*; E & CHV p = 1.00 EKT & EMC p = 1.00; EKT & C p < 0.01* EKT & CHV p = 1.00; EMC & C p < 0.01* EMC & CHV p = 1.00; C & CHV p < 0.01*				
Left	E	22.49 (19.28–25.70)	7.60	23.35 (16.45–28.35)	8.10–34.40	20.31 (17.48–23.14)	6.70	21.70 (15.40–24.65)	7.90–32.80	p = 0.03* (0.42)
	EKT	26.82 (23.45–30.19)	6.77	27.05 (21.80–32.50)	12.30–38.50	24.83 (21.40–28.27)	6.91	24.30 (21.70–29.10)	12.00–38.00	p = 0.01* (0.46)
	EMC	23.65 (20.30–27.66)	6.33	24.30 (18.30–29.60)	12.70–33.90	22.13 (18.13–26.13)	7.22	22.20 (19.00–27.50)	5.30–36.40	p = 0.13 (0.40)
	C	6.27 (5.23–6.87)	1.99	6.10 (4.60–8.00)	1.90–9.00	6.35 (5.55–7.13)	2.16	6.30 (5.40–8.50)	1.50–9.00	p = 0.75 (0.09)
	CHV	17.88 (14.72–21.43)	8.14	16.50 (12.60–23.60)	1.10–35.00	18.50 (15.13–22.11)	8.40	18.20 (12.10–23.90)	2.40–35.00	p = 0.23 (0.09)
	E & EKT p = 0.19; E & EMC p = 0.98 E & C p < 0.01*; E & CHV p = 0.09 EKT & EMC p = 0.61; EKT & C p < 0.01* EKT & CHV p < 0.01*; EMC & C p < 0.01* EMC & CHV p = 0.05; C & CHV p < 0.01*					E & EKT p = 1.00; E & EMC p = 1.00 E & C p < 0.01*; E & CHV p = 1.00 EKT & EMC p = 1.00; EKT & C p < 0.01* EKT & CHV p = 0.22; EMC & C p < 0.01* EMC & CHV p = 1.00; C & CHV p < 0.01*				

CI–confidence interval; EF - Effect Size dppc2 sensu Morris (Morris, S. B. (2008). *Estimating Effect Sizes from Pretest-Posttest-Control Group Designs*. organizational research methods, 11 (2), 364–386. http://doi.org/10.1177/1094428106291059).

E–women with hallux valgus doing corrective exercises; EKT, women with hallux valgus doing corrective exercises and wearing Kinesio tape; EMC, women with hallux valgus performing corrective exercises and using an orthosis at night; CHV, control group, women with hallux valgus; * - statistically significant difference.

Between the first and second examinations, the β angle decreased significantly in group E and increased significantly in group C. Despite these changes, no intergroup differences were noted in either the first or second examinations (Table 3).

**TABLE 3:** Variation in the angle of the little toe (β) of the right and left feet in women under study before and after therapy.

Foot	Group	1st term of study				2nd term of study				p^1st & 2nd^ (EF)
		x̅ (CI)	SD	Me (Q1-Q3)	Min-Max	x̅ (CI)	SD	Me (Q1-Q3)	Min–Max	
Right	E	15.51 (13.13–17.89)	5.64	16.80 (10.60–19.75)	5.90–24.90	12.90 (10.89–14.90)	4.74	11.75 (9.00–15.60)	5.00–24.90	p < 0.01* (0.67)
	EKT	15.11 (12.10–18.12)	6.06	13.50 (10.80–20.50)	6.40–28.20	12.99 (10.32–15.66)	5.37	14.60 (10.10–17.00)	2.10–21.70	p = 0.18 (0.56)
	EMC	16.22 (13.53–20.33)	4.93	15.80 (10.60–21.00)	5.60–23.80	14.13 (11.76–19.20)	5.63	15.40 (10.20–19.30)	1.70–23.80	p = 0.22 (0.62)
	C	15.45 (13.96–16.94)	4.06	15.70 (12.70–18.60)	5.40–22.20	16.11 (14.35–17.87)	4.79	16.80 (12.60–20.60)	3.70–22.80	p = 0.28 (0.16)
	CHV	15.25 (13.05–17.77)	5.70	17.20 (10.10–19.70)	5.40–24.30	16.71 (14.15–22.47)	5.59	18.00 (13.20–19.70)	1.90–26.90	p = 0.17 (0.16)
	E & EKT p = 1.00; E & EMC p = 0.92 E & C p = 1.00; E & CHV p = 1.00 EKT & EMC p = 0.85; EKT & C p = 1.00 EKT & CHV p = 1.00; EMC & C p = 0.89 EMC & CHV p = 0.86; C & CHV p = 1.00					E & EKT p = 1.00; E & EMC p = 0.58 E & C p = 0.18; E & CHV p = 0.10 EKT & EMC p = 0.67; EKT & C p = 0.29 EKT & CHV p = 0.17; EMC & C p = 1.00 EMC & CHV p = 0.96; C & CHV p = 0.99				
Left	E	14.80 (12.51–17.09)	5.43	14.60 (12.25–18.40)	1.20–23.90	14.71 (11.92–17.50)	6.61	12.60 (9.95–19.20)	4.20–27.10	p = 0.94 (0.38)
	EKT	16.82 (14.21–19.44)	5.26	17.05 (13.40–20.80)	7.40–25.20	15.39 (12.40–18.39)	6.03	14.45 (10.80–19.60)	4.40–30.20	p = 0.28 (0.68)
	EMC	17.01 (13.36–18.94)	6.13	16.90 (13.10–20.80)	8.20–29.70	15.48 (11.01–17.25)	6.71	14.70 (11.00–18.20)	5.60–29.70	p = 0.15 (0.66)
	C	16.22 (14.68–17.75)	4.18	16.20 (13.30–18.60)	9.10–24.20	17.97 (16.33–19.61)	4.47	18.60 (14.00–21.80)	10.10–25.70	p < 0.01* (0.38)
	CHV	15.63 (14.16–17.47)	4.15	15.60 (13.00–18.50)	5.90–25.40	15.74 (13.43–18.05)	5.60	17.10 (11.80–18.90)	6.40–26.20	p = 0.89 (0.38)
	E & EKT p = 0.65; E & EMC p = 0.89 E & C p = 0.81; E & CHV p = 0.97 EKT & EMC p = 1.00; EKT & C p = 0.99 EKT & CHV p = 0.93; EMC & C p = 1.00 EMC & CHV p = 1.00; C & CHV p = 0.99					E & EKT p = 1.00; E & EMC p = 1.00 E & C p = 0.21; E & CHV p = 0.97 EKT & EMC p = 0.97; EKT & C p = 0.54 EKT & CHV p = 1.00; EMC & C p = 0.20 EMC & CHV p = 0.91; C & CHV p = 0.58				

CI–confidence interval; EF - Effect Size dppc2 sensu Morris (Morris, S. B. (2008). *Estimating Effect Sizes from Pretest-Posttest-Control Group Designs*. organizational research methods, 11 (2), 364–386. http://doi.org/10.1177/1094428106291059).

E–women with hallux valgus doing corrective exercises; EKT, women with hallux valgus doing corrective exercises and wearing Kinesio tape; EMC, women with hallux valgus performing corrective exercises and using an orthosis at night; CHV, control group, women with hallux valgus; * - statistically significant difference.

At the first examination, the Wejsflog index was significantly greater in group C than in all groups of women with hallux valgus. After 12 weeks, under the influence of therapy, the Wejsflog index increased significantly in group E in the right foot and in group EKT in the left foot. However, at the second examination, the Wejsflog index was still significantly lower in both of these groups than in the group of women with properly shaped feet (Table 4).

**TABLE 4:** The value of the Wejsflog index for the right and left feet of women before and after therapy.

Foot	Group	1st term of study				2nd term of study				p^1st & 2nd^ (EF)
		x̅ (CI)	SD	Me (Q1-Q3)	Min-Max	x̅ (CI)	SD	Me (Q1-Q3)	Min–Max	
Right	E	2.28 (2.22–2.34)	0.13	2.29 (2.20–2.37)	2.02–2.48	2.31 (2.25–2.37)	0.14	2.33 (2.21–2.41)	2.03–2.58	p = 0.02* (0.34)
	EKT	2.25 (2.17–2.31)	0.14	2.25 (2.21–2.34)	1.97–2.50	2.26 (2.19–2.34)	0.15	2.25 (2.20–2.36)	1.97–2.52	p = 0.17 (0.17)
	EMC	2.24 (2.16–2.32)	0.14	2.25 (2.14–2.33)	1.98–2.51	2.26 (2.18–2.34)	0.16	2.26 (2.16–2.37)	1.93–2.51	p = 0.18 (0.25)
	C	2.45 (2.41–2.49)	0.10	2.45 (2.38–2.51)	2.25–2.69	2.44 (2.40–2.48)	0.11	2.43 (2.38–2.51)	2.22–2.66	p = 0.23 (0.01)
	CHV	2.30 (2.25–2.36)	0.13	2.27 (2.22–2.43)	2.09–2.56	2.29 (2.24–2.35)	0.12	2.28 (2.22–2.40)	2.10–2.56	p = 0.34 (0.01)
	E & EKT p = 0.82; E & EMC p = 0.92 E & C p < 0.01*; E & CHV p = 0.97 EKT & EMC p = 1.00; EKT & C p < 0.01* EKT & CHV p = 0.47; EMC & C p < 0.01* EMC & CHV p = 0.64; C & CHV p < 0.01*					E & EKT p = 0.73; E & EMC p = 0.68 E & C p < 0.01*; E & CHV p = 0.97 EKT & EMC p = 1.00; EKT & C p < 0.01* EKT & CHV p = 0.96; EMC & C p < 0.01* EMC & CHV p = 0.93; C & CHV p < 0.01*				
Left	E	2.30 (2.24–2.36)	0.15	2.28 (2.21–2.42)	1.99–2.63	2.30 (2.25–2.36)	0.14	2.31 (2.24–2.40)	1.94–2.54	p = 0.66 (0.01)
	EKT	2.25 (2.19–2.32)	0.12	2.28 (2.12–2.33)	2.03–2.40	2.29 (2.22–2.36)	0.12	2.31 (2.14–2.30)	2.04–2.48	p = 0.01* (0.32)
	EMC	2.21 (2.14–2.27)	0.13	2.19 (2.17–2.37)	1.99–2.45	2.21 (2.15–2.28)	0.13	2.21 (2.19–2.40)	1.92–2.45	p = 0.69 (0.01)
	C	2.50 (2.46–2.55)	0.12	2.49 (2.42–2.56)	2.25–2.83	2.50 (2.45–2.54)	0.12	2.49 (2.42–2.55)	2.25–2.81	p = 0.45 (0.01)
	CHV	2.33 (2.27–2.38)	0.13	2.33 (2.22–2.41)	2.10–2.63	2.33 (2.26–2.38)	0.14	2.36 (2.22–2.39)	2.10–2.63	p = 1.00 (0.01)
	E & EKT p = 0.17; E & EMC p = 0.86 E & C p < 0.01*; E & CHV p = 0.95 EKT & EMC p = 0.82; EKT & C p < 0.01* EKT & CHV p = 0.03*; EMC & C p < 0.01* EMC & CHV p = 0.47; C & CHV p < 0.01*					E & EKT p = 0.17; E & EMC p = 1.00 E & C p < 0.01*; E & CHV p = 0.98 EKT & EMC p = 0.45; EKT & C p < 0.01* EKT & CHV p = 0.05; EMC & C p < 0.01* EMC & CHV p = 0.91; C & CHV p < 0.01*				

CI–confidence interval; EF - Effect Size dppc2 sensu Morris (Morris, S. B. (2008). *Estimating Effect Sizes from Pretest-Posttest-Control Group Designs*. organizational research methods, 11 (2), 364–386. http://doi.org/10.1177/1094428106291059).

E–women with hallux valgus doing corrective exercises; EKT, women with hallux valgus doing corrective exercises and wearing Kinesio tape; EMC, women with hallux valgus performing corrective exercises and using an orthosis at night; CHV, control group, women with hallux valgus; * - statistically significant difference.

The qualitative assessment of the transverse arch, estimated using the Wejsflog index, showed that in the first examination, the transverse arch of the right foot was normal in three women with hallux valgus (one from the EKT group, one from the EMC group and one from the CHV group) and ten women with the correct position of hallux valgus. After 12 weeks, nine women from group C and seven women with hallux valgus had normal right foot arches (two from group E, two from group EKT, one from group EMC and two from group CHV).

In the left foot, in the first examination, five women with hallux valgus (two from group E and three from group CHV) and 14 women with normal hallux valgus had a normal transverse arch. After 12 weeks, 15 women from group C and six women with hallux valgus (two from group E and four from group CHV) had a normal left foot arch.

Clarke’s angle did not differ between the groups of women with hallux valgus and those with a normal foot shape at either the first or second examination. The applied therapy did not change Clarke’s angle in any of the groups (Table 5).

**TABLE 5:** The value of Clarke’s angle for the right and left feet in women under research before and after therapy.

Foot	Group	1st term of study				2nd term of study				p^1st & 2nd^ (EF)
		x̅ (CI)	SD	Me (Q1-Q3)	Min-Max	x̅ (CI)	SD	Me (Q1-Q3)	Min–Max	
Right	E	46.47 (43.49–49.44)	7.05	46.80 (45.05–50.85)	30.40–60.60	46.47 (44.32–48.62)	5.08	46.15 (42.50–49.25)	37.80–58.50	p = 0.95 (0.08)
	EKT	45.93 (42.88–48.97)	6.12	45.45 (41.70–51.20)	33.70–56.40	46.54 (42.76–50.31)	7.59	47.90 (42.00–51.20)	31.30–63.70	p = 0.68 (0.01)
	EMC	47.32 (43.73–51.28)	6.88	48.30 (43.10–51.30)	34.80–62.90	46.96 (43.74–50.82)	5.99	46.60 (44.00–50.40)	37.20–61.20	p = 0.70 (0.16)
	C	43.51 (41.84–45.19)	4.56	43.20 (39.30–47.10)	36.30–53.60	44.03 (42.58–45.88)	4.35	43.80 (41.00–47.90)	36.80–54.20	p = 0.08 (0.14)
	CHV	43.67 (41.14–46.21)	6.44	44.70 (40.30–47.90)	33.50–56.30	43.40 (40.70–46.10)	6.55	43.60 (38.00–47.80)	32.40–56.30	p = 0.78 (0.14)
	E & EKT p = 1.00; E & EMC p = 1.00 E & C p = 0.34; E & CHV p = 0.56 EKT & EMC p = 1.00; EKT & C p = 1.00 EKT & CHV p = 1.00; EMC & C p = 0.46 EMC & CHV p = 0.66; C & CHV p = 1.00					E & EKT p = 1.00; E & EMC p = 1.00 E & C p = 0.54; E & CHV p = 0.35 EKT & EMC p = 1.00; EKT & C p = 0.59 EKT & CHV p = 0.41; EMC & C p = 0.50 EMC & CHV p = 0.34; C & CHV p = 0.99				
Left	E	44.19 (41.54–46.83)	6.27	44.35 (42.00–48.60)	31.00–54.00	43.50 (40.83–46.18)	6.33	44.50 (40.75–48.30)	37.80–58.50	p = 0.48 (0.03)
	EKT	43.07 (39.77–46.38)	6.64	43.45 (41.20–46.50)	25.80–54.10	43.41 (41.35–45.47)	4.14	44.35 (40.20–46.20)	31.30–63.70	p = 0.79 (0.12)
	EMC	44.18 (41.54–46–83)	6.97	45.00 (38.00–49.30)	33.90–59.30	44.47 (40.83–46.18)	7.02	43.90 (40.80–47.80)	37.20–61.20	p = 0.73 (0.11)
	C	43.99 (41.83–46.15)	5.89	44.10 (39.70–48.80)	31.50–58.10	43.54 (41.36–45.72)	5.95	43.90 (39.50–46.50)	36.80–54.20	p = 0.27 (0.06)
	CHV	43.58 (41.02–46.13)	6.18	43.90 (39.90–47.80)	30.00–58.20	43.51 (40.85–46.17)	6.44	44.20 (39.80–46.90)	32.40–56.30	p = 0.85 (0.06)
	E & EKT p = 0.98; E & EMC p = 1.00 E & C p = 1.00; E & CHV p = 1.00 EKT & EMC p = 0.99; EKT & C p = 0.99 EKT & CHV p = 1.00; EMC & C p = 1.00 EMC & CHV p = 1.00; C & CHV p = 1.00					E & EKT p = 1.00; E & EMC p = 0.99 E & C p = 1.00; E & CHV p = 1.00 EKT & EMC p = 0.99; EKT & C p = 1.00 EKT & CHV p = 1.00; EMC & C p = 0.99 EMC & CHV p = 0.99; C & CHV p = 1.00				

CI–confidence interval; EF - Effect Size dppc2 sensu Morris (Morris, S. B. (2008). *Estimating Effect Sizes from Pretest-Posttest-Control Group Designs*. organizational research methods, 11 (2), 364–386. http://doi.org/10.1177/1094428106291059).

E–women with hallux valgus doing corrective exercises; **EKT**, women with hallux valgus doing corrective exercises and wearing Kinesio tape; **EMC**, women with hallux valgus performing corrective exercises and using an orthosis at night; **CHV**, control group, women with hallux valgus; * - statistically significant difference.

In the first examination, the correct longitudinal arch of the right foot, estimated based on Clarke’s angle, was determined in 17 women in the normal hallux position, 19 in group E, 14 in group EKT, 11 in group EMC and 14 in group CHV. In the left foot, at the first examination, a normal longitudinal arch was observed in 20 women in the normal hallux position, 17 in group E, 14 in group EKT, 16 in group EMC and 16 in group CHV. After 12 weeks, the number of women with normal longitudinal arches changed only in group E, where two women had lower longitudinal arches in both feet.

## Discussion

Each of the therapy programmes applied significantly reduced the pain in the large toe area. There was also a decrease in the hallux valgus angle (a significant change in the group of women performing exercises and those performing exercises combined with kinesiology taping) and a decrease in the little toe varus angle (a significant change in the group of women performing exercises). There were no differences in the height of the longitudinal arch of the foot (estimated using the Clarke angle) between women with hallux valgus and those with a normal hallux position, nor any changes in the longitudinal arch of the foot under the influence of therapy. Women with hallux valgus had significantly lower Wejsflog index values, which is related to the greater width of the forefoot and may indirectly indicate a lower transverse arch of the foot. The effectiveness of exercises in the treatment of hallux valgus has been confirmed by other authors. In elite teenage classical dancers, a 4-week foot exercise program reduced the hallux valgus angle (from 20.1° to 15.4°) and pain, and improved foot function during demi-pointe and plié dance techniques (plantar pressure was measured in six areas of the foot during the performance of dance techniques) (Liu et al., 2024). Our study results indicate that supplementing the exercise programme with kinesiology taping or using an orthosis at night is not justified because it does not significantly increase the effect of the therapy.

The effectiveness of exercises combined with kinesiology taping on the pain and alignment of hallux valgus was also studied by Bayar et al. (2011). Their observations showed that the combination of taping with exercise had a greater impact on the hallux valgus angle and foot pain than the exercise itself. However, it should be emphasized that the cited authors conducted therapy for a shorter period of time (8 weeks), and the exercise programme they used was much less intense (patients performed two exercises twice a day—passive and active hallux abduction—with each exercise repeated ten times. Additionally, the exercises were performed without the supervision of a physiotherapist, and there was no information on their regularity). The advantage of the study was that it assessed pain both at rest and while walking. In turn, Saikhanzul et al. (2022) compared the effectiveness of 4 weeks of Bosu® ball exercises to 4 weeks of kinesiology taping on hallux valgus pain and angle in young adults. The results showed that both therapy methods had a significant and similar effect on the tested parameters.

It is difficult to find studies that compare the effectiveness of the treatment of hallux valgus with that of exercise to that of therapy using mechanical correction with the help of orthoses. Abdalbary (2018) used the American Orthopedic Foot and Ankle Society (AOFAS) scale to compare women with moderate hallux valgus who, over 3 months, had 36 therapeutic sessions, which included mobilization of the ankle joint, exercises for the plantar flexion and abduction of the hallux, exercises increasing toe grip strength, and exercises increasing the range of dorsiflexion of the ankle joint; throughout this period, the use of a toe separator by women with hallux valgus did not undergo therapy. The authors performed a number of objective measurements—the ankle’s range of motion, plantar flexion and abduction strength, and toe grip strength—using radiographic angular measurements and the AOFAS scale. There was a reduction in pain intensity, improvement in functional performance and radiographic measurements, and an increase in plantar flexion and hallux abduction strength, as well as toe grip strength and dorsiflexion range immediately after treatment and after 1 year of follow-up. Unfortunately, this study does not provide information about whether the same set of exercises without the use of a toe separator was equally or less effective. Kim MH. et al. (2015) observed changes in the hallux valgus angle and the cross-section of the hallux abductor muscle using X-rays and ultrasound in a group of patients who underwent mechanical correction with orthosis and in a group of patients who, in addition to wearing an orthosis, performed toe abduction exercises (spread-out). In both, the intervention lasted 8 weeks. While the exercises reduced the hallux valgus angle and increased the cross-sectional area of the hallux abductor muscle, no significant changes were noted in the group of patients who used orthoses. Researchers agree that dynamic orthoses are more effective in eliminating pain and reducing the hallux valgus angle than static orthoses. Dynamic orthoses are also better tolerated by patients in everyday functioning. Orthoses and separators should be used systematically for several months, 6–8 h each day, to achieve a long-term effect (Pawłowski et al., 2024).

The discussion about the transverse arch of the foot currently causes much controversy. Although anthropological research has sufficiently proven that the development of the proximal transverse arch during the evolution of hominids was crucial for the effective mechanism of pushing off the ground while walking (Venkadesan et al., 2020), the existence of the distal transverse arch is no longer as obvious. Luger et al. (1999) argued that the heads of the five metatarsal bones form a transverse arch that helps load transmission and shock absorption and thus facilitates propulsion, but other authors deny this thesis, citing the results of foot pressure measurements on a sensory platform or biomechanical analysis (Metera and Saran, 2018; Zeidan et al., 2020). Regardless of these considerations on the anatomy and biomechanics of the foot, one cannot ignore the clinical observation about the repeatedly confirmed relationship between the occurrence of hallux valgus and an increase in the width of the foot interpreted (perhaps incorrectly?) as a decrease in the transverse arch (Chen et al., 2022; Jankowicz-Szymańska et al., 2022; Nakai et al., 2019). The mechanism of loading the forefoot during foot roll is probably of key importance here. Specifically, 40% of the body weight should be rolled over the metatarsophalangeal joint of the hallux, but its incorrect positioning in the case of valgus increases the load on the remaining metatarsal bones, which causes overloading and pain under the heads of the middle metatarsals (DiGiovanni and Greisberg, 2017). In this study, a greater width of the forefoot was also observed in women with hallux valgus, and the width of the forefoot tended to decrease under the influence of the therapy used. There was also a tendency (not confirmed to be statistically significant except in one patient) to decrease the varus angle of the little toe under the influence of therapy in each group. Interestingly, Puszczalowska-Lizis et al. (2022) also reported a relationship between lowering of the transverse arch and varus deformity of the little toe in preschool children. Similar observations were made by Mikuľáková et al. (2020), who examined healthy students. Nearly half of the respondents were diagnosed with a lower transverse arch of the foot (they also used the Wejsflog index), more than one-third had a fifth toe varus, and half, mostly women, had hallux valgus.

Many researchers agree about the longitudinal arch in people with hallux valgus. Similarly, Marek et al. (2019) reported that most older adult women with hallux valgus have normal longitudinal foot arches. Our study also revealed no correlation between hallux valgus and the longitudinal arch of the foot.

The limitation of our study is that only volunteers qualified for therapy, which means that the results should be applied cautiously to the general population. The authors are aware that considering the controversy related to the transverse arch of the foot, conclusions about its height based on the study of changes in the width of the forefoot are far from perfect. However, the Wejsflog index was used because this index has been commonly used for many decades (Wejsflog, 1955). The main limitation of the study is assessment after 12 weeks of applied treatment without longer follow-up. The longer follow-up and effectiveness are unknown.

We hope our study will contribute new evidence to the scientific discussion about the deformity of hallux valgus. Moreover, we want to draw attention to the practical aspects of this study. Properly conducted exercises significantly benefit patients with hallux valgus, and the introduction of additional interventions in the form of kinesiology taping or orthotics may be an unnecessary economic cost that can be ignored.

## Conclusion

1. Hallux valgus in women is associated with a larger forefoot width, which may indirectly indicate a lowered transverse arch of the foot, while the height of the longitudinal arch does not differ between women with hallux valgus and those with a normal hallux position.
2. 12-weeks of conservative treatment can significantly reduce foot pain in women with hallux valgus. However, it should be remembered that the beneficial effects of therapy were noted during its duration, and it was not observed whether they would be maintained in the long term.
3. 12-weeks of conservative treatment methods, particularly exercises and exercises combined with kinesiology taping, significantly improve the alignment of the hallux and little toe and reduce the width of the forefoot.
4. Supplementing the exercise programme with kinesiology taping or using an orthosis at night does not significantly increase the effect of therapy.

## Data Availability

The raw data supporting the conclusions of this article will be made available by the authors, without undue reservation.
